# ﻿A review of the subgenus *Loxocera* Meigen, 1803 (Diptera, Brachycera, Psilidae) in China

**DOI:** 10.3897/zookeys.1186.108876

**Published:** 2023-12-11

**Authors:** Jiale Zhou, Ding Yang

**Affiliations:** 1 Department of Entomology, College of Plant Protection, China Agricultural University, 2 Yuanmingyuan West Road, Beijing 100193, China China Agricultural University Beijing China

**Keywords:** Acalyptratae, Diopsoidea, *Imantimyia*, new species, Palaearctic Realm, Psilinae

## Abstract

The subgenus Loxocera (*Loxocera* Meigen, 1803) (Diptera, Brachycera, Psilidae) in China is reviewed. Six species are recognized, including four new species: L. (L.) chikuni**sp. nov.**, L. (L.) lonsdalei**sp. nov.**, L. (L.) maculithorax**sp. nov.**, and L. (L.) obscura**sp. nov.** Two species originally placed in the subgenus Loxocera, *L.anulata* Wang & Yang, 1998 and *L.tianmuensis* Wang & Yang, 1998, are transferred to the subgenus Loxocera (*Imantimyia* Frey, 1925). A key to the species of the subgenus Loxocera occurring in China is provided.

## ﻿Introduction

Psilidae is a group of small to medium-sized, yellow to black acalyptrate flies which can be diagnosed externally by their peculiar wing venation and reduced setation (Lonsdale 2020; Shatalkin 2021). With about 340 species described so far, the Psilidae is distributed in all zoogeographic regions, with the highest diversity occurring in the temperate areas of the northern hemisphere (Shatalkin and Merz 2010; Lonsdale 2020). The monophyly of Psilidae and its several subtaxa are well supported, whereas the generic classification within Psilinae needs further consideration (Buck and Marshall 2006a, 2006b; Lonsdale 2020; Zhou and Yang 2022).

The psiline genus *Loxocera* Meigen, 1803 is cosmopolitan and currently comprises about 50 species. This genus was traditionally divided into four subgenera: *Loxocera* s. str., *Asiopsila* Shatalkin, 1998, *Platystyla* Macquart, 1835, and *Tropeopsila* Shatalkin, 1983 (Shatalkin 1998). Based on a morphological phylogenetic analysis, Buck and Marshall (2006b) redefined the genus, recognized three subgenera (*Loxocera* s. str., *Imantimyia* and *Tropeopsila*), transferred *Asiopsila* to the genus *Psila* Meigen, 1803, and synonymized *Platystyla* with *Loxocera* s. str. The genus *Terarista* Yang & Wang, 2003 has also been confirmed as a junior synonym of the subgenus Loxocera recently (Zhou et al. 2022).

In the present study, we review and key the Chinese fauna of the subgenus Loxocera and document six species, four of which are described here as new. The other two Chinese species previously placed in the subgenus Loxocera are here transferred to *Imantimyia*.

Outside of China, an additional eight species of the subgenus Loxocera are known, with these occurring exclusively within the Palaearctic and northern Oriental realms (Iwasa 1992, 1993; Buck and Marshall 2006b; Zhou et al. 2022). A checklist for all described species of the subgenus Loxocera is also provided in this paper.

## ﻿Materials and methods

Specimens examined in this study are deposited in the Entomological Museum of China Agricultural University, Beijing, China (**CAU**) and the Smithsonian National Museum of Natural History, Washington, DC, USA (**USNM**).

Male terminalia were prepared by macerating the apical portion of the abdomen in heated 10% KOH solution for approximately 10 min, and then rinsing in distilled water. External structure and terminalia were examined using a Nikon SMZ745 stereoscopic microscope. After examination, the terminalia were transferred to fresh glycerol and stored in microvials pinned below the corresponding specimens.

Photographs were taken using a Canon 7D Mark II digital camera with a Canon macro lens EF 100 mm and MP-E 65 mm for habitus, and an Olympus BX51 microscope for terminalia. Figures were stacked using Helicon Focus v. 5.3 and assembled by Adobe Photoshop 2020. The distribution map was prepared using the online version of SimpleMappr (Shorthouse 2010). Terminology follows Buck and Marshall (2006b) and Lonsdale (2020). Measurements were obtained using a calibrated micrometer; body length is measured from apex of frons to apex of abdomen; interocular space is the width between eyes.

## ﻿Results

### ﻿Genus *Loxocera* Meigen, 1803

#### ﻿subgenus Loxocera

Taxon classificationAnimaliaDipteraPsilidae

Meigen, 1803

C7AABB32-BDC9-537C-A94E-F2FFF877CDB1

Loxocera Meigen, 1803: 275. Type species: Muscaaristata Panzer, 1801, by monotypy.
Platystyla Macquart, 1835: 374. Type species: Loxocerahoffmannseggii Meigen, 1826, by monotypy. Synonymized by Buck and Marshall (2006b: 199).
Terarista Wang, 1999: 268. Nomen nudum.
Terarista Yang & Wang in Wang & Yang, 2003: 563. Type species: Teraristafujiana Wang, 1999, by original designation. Synonymized by Zhou et al. (2022: 465).

##### Diagnosis.

The subgenus Loxocera can be recognized by the following combination of character states: frontal vitta desclerotized, dull, velvety; lunule sclerotized, broadly exposed between antennal base and anterior margin of frons; fore wing with alula glabrous except margin; hind femur with a subapical patch of microtomentum on ventral surface; male sternite 8 broadly exposed and setulose, fused to tergite 6 and epandrium; female tergite 10 and cerci separated. For details on the character states used to define the subgenus, see Buck and Marshall (2006b).

### ﻿Key to species of subgenus Loxocera from China

**Table:** 

1	Arista blackish brown, laterally compressed and very high, arising at apex of antennal first flagellomere (Figs 3, 4, 21); wing with broad, transverse, dark band at level of posterior crossvein (Figs 1, 19)	**2**
–	Arista whitish yellow, thin, arising near midpoint of antennal first flagellomere (Figs 12, 29, 36); wing without transverse dark band (Figs 27, 35)	**4**
2	Frontal vitta uniformly black	**L. (L.) fujiana (Wang)**
–	Frontal vitta black with anterior part dark yellow or yellowish brown (Figs 2, 20)	**3**
3	Apex of antennal first flagellomere not produced beyond base of arista (Figs 3, 4); mesonotum blackish brown; hypandrial lobe small, short (Figs 5–8)	**L. (L.) chikuni sp. nov.**
–	Apex of antennal first flagellomere clearly produced beyond base of arista (Fig. 21); mesonotum largely blackish brown, with irregular dark brown margin (Fig. 22); hypandrial lobe large, broad (Figs 23–26)	**L. (L.) maculithorax sp. nov.**
4	Antennal scape and pedicel subequal in length (Fig. 36)	**L. (L.) omei Shatalkin**
–	Antennal scape and pedicel unequal in length (Figs 12, 29)	**5**
5	Antennal scape distinctly shorter than pedicel (Fig. 12); antennal first flagellomere about 3 times as long as pedicel (Fig. 12); fore and mid femora blackish brown with apical half yellowish brown; pregonite short, simple in shape, apically blunt (Fig. 16); phallus shovel-like, abruptly widened apically with rounded posterior margin (Figs 14, 15)	**L. (L.) lonsdalei sp. nov.**
–	Antennal scape distinctly longer than pedicel (Fig. 29); antennal first flagellomere about 4.6 times as long as pedicel (Fig. 29); fore and mid femora dark yellow with base pale yellow, hind femur dark brown; pregonite rather long, slender, curved apically with sharp apex (Fig. 33); phallus droplet-like, abruptly narrowed apically (Figs 31, 32)	**L. (L.) obscura sp. nov.**

#### ﻿ Loxocera (Loxocera) chikuni sp. nov.

Taxon classificationAnimaliaDipteraPsilidae

1ABAA6DE-B020-53ED-80DF-0E911B26B648

https://zoobank.org/48776277-5971-456E-86BD-2EE0265B2A2D

Figs 1–4 , 5–8

##### Type material.

***Holotype*** (♂): China, Hubei, Shennongjia, Guanmenshan, 1560 m, 2019.viii.12, leg. Ding Yang (CAU).

##### Diagnosis.

Generally blackish brown; face blackish; antennal scape and pedicel subequal in length; apex of antennal first flagellomere not produced beyond base of arista; arista laterally compressed and very high, arising at apex of first flagellomere and 1.8 times as long as the latter; wing with broad, transverse, dark band at level of posterior crossvein; hypandrial lobe small, short, covered with long setae; pregonite stout, lobate, apically blunt with rounded processes; phallus tongue-like, relatively short, with rounded posterior margin.

##### Description.

**Figures 1–4.**
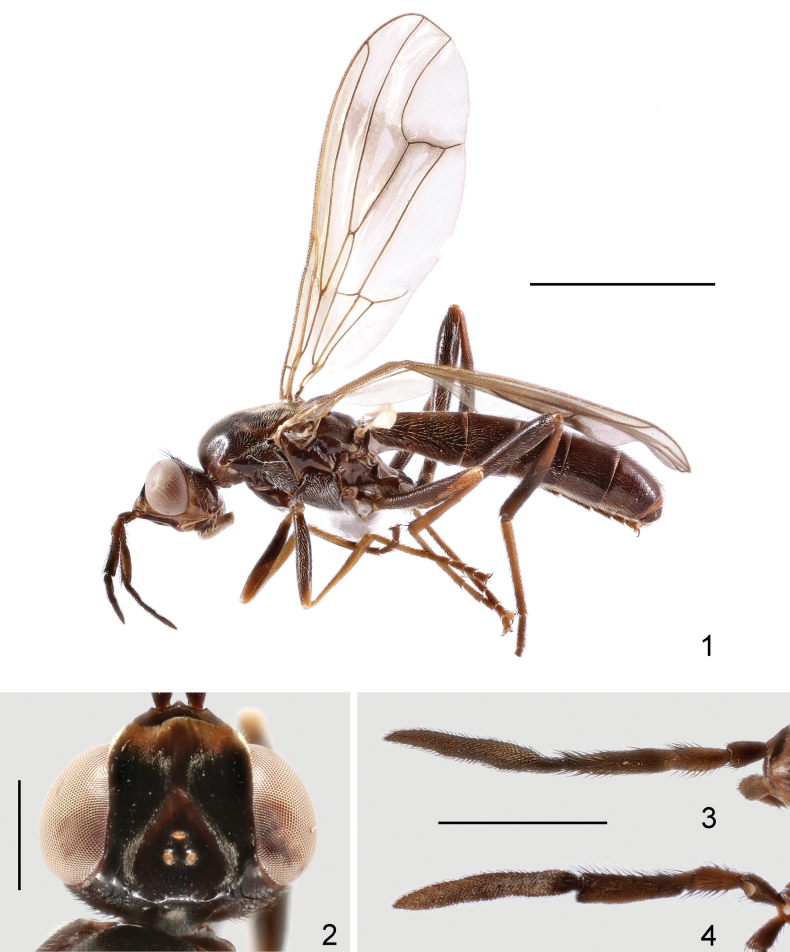
Loxocera (Loxocera) chikuni sp. nov., holotype, male **1** habitus, lateral **2** head, dorsal **3** left antenna, dorsal **4** same, lateral. Scale bars: 3 mm (**1**); 0.5 mm (**2**); 1 mm (**3, 4**).

**Male.** Body length 9.0 mm, wing length 7.2 mm, length of antenna 2.5 mm. Generally blackish brown, moderately shining (Fig. 1). Frontal vitta black, with anterior part yellowish brown (Fig. 2); parafacial, gena and posterior eye margin yellowish brown; proboscis and palpus pale brown. Wing slightly infumated, with broad, transverse, dark band at level of posterior crossvein, band more or less interrupted along center of cells r_4+5_ and dm (Fig. 1); wing veins yellowish brown to brown. Halter white with base slightly darkened. Legs with trochanters, apical half of fore and mid femora, fore and mid tibiae, basal half of hind tibia, and tarsomere I pale brown. Bristles on head and thorax black.

Head (Figs 1, 2) transverse oblong in dorsal view, largely glabrous; length along midline 0.8 times as long as width across eyes, width across eyes 1.9 times as broad as interocular space. Frons slightly protruding beyond level of anterior eye margin; frontal vitta with shallow depression at middle; frontal orbit with some short, scattered hairs. Ocellar triangle broad, smooth, with silvery tomentose stripes along lateral margins. Face strongly slanting, with weakly elevated median carina. Parafacial narrow, with a tomentose golden patch between anterior eye margin and lunule. Gena swollen; postgena covered with silvery tomentum. Occiput with a large silvery tomentose patch at middle above foramen. Head chaetotaxy: 1 ocellar seta, 1 postvertical seta, 1 inner vertical seta, 1 outer vertical seta. Antenna (Figs 3, 4) long and thick, with short dense setulae; scape nearly as long as pedicel, gradually widened towards apex of segment; first flagellomere about 2.1 times as long as pedicel, apex very weakly curving ventrally; arista laterally compressed and very high, arising at apex of first flagellomere, 1.8 times as long as first flagellomere, divided into small aristomere 1 and large aristomeres 2+3. Palpus elongate oval, with short, dense setulae and long, scattered setae.

Thorax (Fig. 1) robust, with short, dense, whitish-yellow setulae, except anepisternum (anterior half), anepimeron, katatergite, meron, scutellum and mediotergite (middle portion) glabrous; anatergite with fine tomentum; disc of scutellum with fine wrinkles. Scutum 1.4 times as long as wide. Scutellum subtriangular, slightly swollen, and wider than long. Thoracic chaetotaxy: 1 dorsocentral seta, 1 notopleural seta, 2 posterior supra-alar setae, 1 apical scutellar seta. Wing (Fig. 1) with last sector of M_1_ strongly curved; apex of M_4_ nearly reaching wing margin. Legs with dense, whitish-yellow setulae, except ventral surface of fore and mid femora largely glabrous; femora subfusiform, slightly compressed laterally; tibiae gradually widened towards apex, fore and mid tibiae straight, hind tibia finely curved.

Abdomen elongate, with short, dense, whitish-yellow and black setae; syntergite 1+2 with several long, hair-like setae laterally.

**Figures 5–8.**
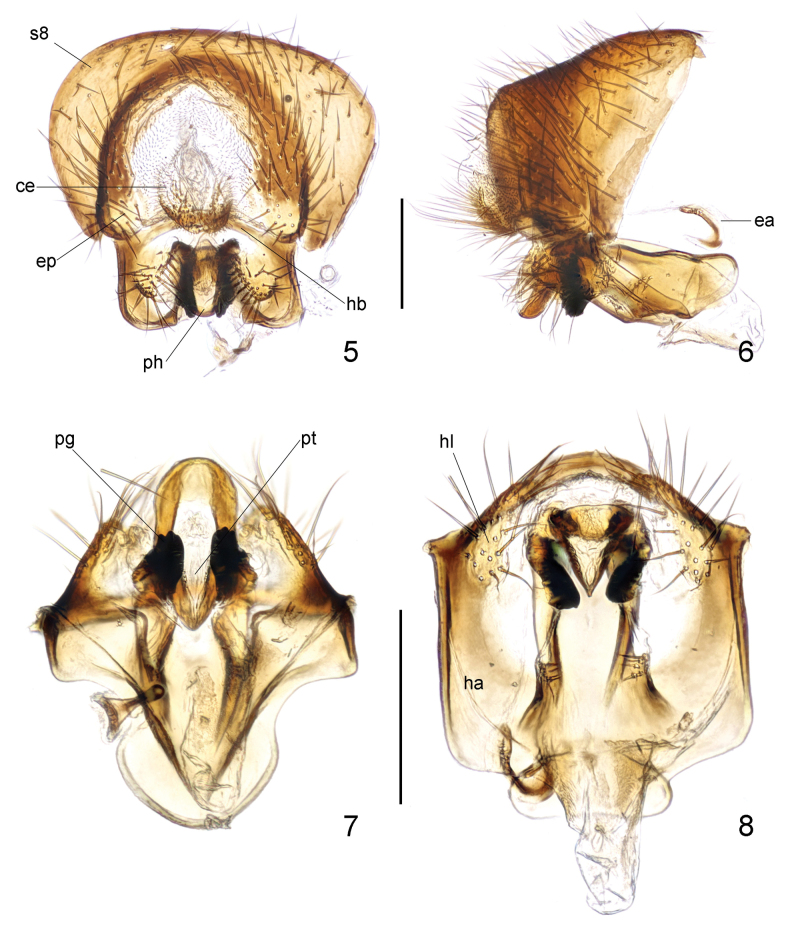
Loxocera (Loxocera) chikuni sp. nov., male genitalia **5** sternite 8 to genitalia, caudal **6** same, lateral **7** pregonite and phallus, ventral **8** hypandrium and associated structures, ventral. Abbreviations: ce = cercus, ea = ejaculatory apodeme, ep = epandrium, ha = hypandrial arm, hb = hypandrial bridge, hl = hypandrial lobe, pg = pregonite, s8 = sternite 8, ph = phallus, pt = phallotrema.Scale bar: 0.25 mm.

***Male genitalia***: Sternite 8 (Figs 5, 6) broad, relatively flattened, with long, dense setae. Cerci (Figs 5, 6) small, simple in shape, with short, dense setae. Hypandrium (Fig. 8) well developed; hypandrial arms fused posterodorsally, posteriorly produced into small, short, convex lobes covered with long setae (Figs 5–8). Hypandrial bridge present and robust. Pregonite (Figs 5–7) stout, lobate, apically blunt, with some rounded processes. Phallus (Figs 5–7) tongue-like, relatively short, with rounded posterior margin. Phallotrema (Fig. 7) large, flanked by some peculiar, tiny processes. Ejaculatory apodeme (Fig. 6) small, hook-like, strongly curved.

**Female.** Unknown.

##### Etymology.

The specific epithet is dedicated to the Chinese entomologist Chikun Yang (1925–2006), in honor of his excellent contribution to Chinese insect taxonomy.

##### Distribution.

China – Hubei: Shennongjia (Fig. 37).

##### Comparative notes.

This new species is similar to L. (L.) fujiana (Wang, 1999) and L. (L.) maculithorax sp. nov. by sharing the laterally compressed and high arista which arises at the apex of the antennal first flagellomere, and the broad, transverse, dark band on the wing. The new species differs from L. (L.) fujiana by the following character states: head blackish brown with anterior part of frontal vitta, parafacial, gena, and posterior eye margin yellowish brown [vs head blackish brown with gena slightly paler in L. (L.) fujiana]; arista 1.8 times as long as antennal first flagellomere [vs 2 times in L. (L.) fujiana]; hind tibia pale brown in basal half and blackish brown in apical half [vs uniformly dark brown in L. (L.) fujiana]; transverse dark band on wing interrupted along cell r_2+3_ [vs not interrupted in cell r_2+3_ in L. (L.) fujiana]. The new species can be separated from L. (L.) maculithorax sp. nov. by the following character states: apex of antennal first flagellomere not produced beyond base of arista [vs produced beyond base of arista in L. (L.) maculithorax sp. nov.]; arista 1.8 times as long as antennal first flagellomere [vs 2.1 times in L. (L.) maculithorax sp. nov.]; mesonotum uniformly blackish brown [vs largely blackish with irregular brownish margin in L. (L.) maculithorax sp. nov.]; abdomen uniformly blackish brown [vs reddish brown with both ends darkened in L. (L.) maculithorax sp. nov.]; male sternite 8 flattened [vs with a small blunt protrusion in L. (L.) maculithorax sp. nov.]; hypandrial lobe of male small and short [vs large and broad in L. (L.) maculithorax sp. nov.].

#### ﻿ Loxocera (Loxocera) fujiana

Taxon classificationAnimaliaDipteraPsilidae

(Wang, 1999)

E6795CC1-50BE-54D0-9E2A-5CE17318B9D2

Terarista fujiana Wang, 1999: 268 (protologue); Wang and Yang (2003: 563, 565) (subsequent usage, redescription, lectotype designation, figure); Cui et al. (2009: 326) (listed); Liang (2016: 170) (listed); Tang et al. (2021: 236) (catalogue, distribution). Lectotype (♀): China, Fujian, Wuyishan, CAU.
Loxocera (Loxocera) fujiana : Zhou et al. (2022: 465) (new combination, redescription, distribution, photo).

##### Type material examined.

***Lectotype*** (♀): China, Fujian, Wuyishan, Guadun, 1991.x.7, leg. Hong Wu (CAU).

##### Diagnosis.

Generally blackish brown; face black; antennal scape and pedicel subequal in length; apex of antennal first flagellomere not produced beyond base of arista; arista laterally compressed and very high, arising at apex of antennal first flagellomere and 2 times as long as the latter; wing with broad, transverse, dark band at level of posterior crossvein; segment 8 of female with shallowly emarginated posterodorsal margin and deeply incised posteroventral margin.

##### Distribution.

China – Fujian: Wuyishan (Fig. 37).

##### Remarks.

For redescription and photographs of this species, see Zhou et al. (2022).

#### ﻿ Loxocera (Loxocera) lonsdalei sp. nov.

Taxon classificationAnimaliaDipteraPsilidae

0B2487E1-16E4-53B0-B3F8-EE220FBA7E38

https://zoobank.org/78375471-5312-46F1-89EB-59C7BDA7DAA2

Figs 9–10 , 11–13 , 14–17

##### Type materials.

***Holotype*** (♂): China, Shaanxi, Xi’an, Huyi, Zhuque Forest Park, 2606 m, 2020.vii.10, leg. Bing Zhang (CAU). ***Paratypes***: China, Qinghai, Haibei, Menyuan, Deqian vill., 2725 m, 2019.vii.18, leg. Jinlong Ren (1♀, CAU); China, Qinghai, Haidong, Huzhu, Beishan Forest Farm, Zhalonggou, 2724 m, 2019.vii.2, leg. Qilemoge (1♂1♀, CAU); CHINA, Qinghai, Haidong, Huzhu, Yuanfugou, 2682 m, 2019.vii.3, leg. Qilemoge (1♀, CAU); same collection data as for holotype (5♂5♀, CAU); China, Shaanxi, Xi’an, Zhouzhi, Wangjiahe, 1165 m, 2020.vii.6, leg. Bing Zhang (1♀, CAU).

##### Diagnosis.

Generally brown to blackish brown; face yellowish brown; antennal pedicel distinctly longer than scape; antennal first flagellomere about 3 times as long as pedicel, gradually narrowed towards apex; arista whitish yellow, thin, arising near midpoint of antennal first flagellomere and 1.3 times as long as the latter; mesonotum blackish, with irregular brownish margin; wing without transverse dark band; hypandrial lobe large, broad, covered with short setae; pregonite short, simple in shape, apically blunt; phallus shovel-like, relatively long, abruptly widened apically with rounded posterior margin.

##### Description.

**Figures 9, 10.**
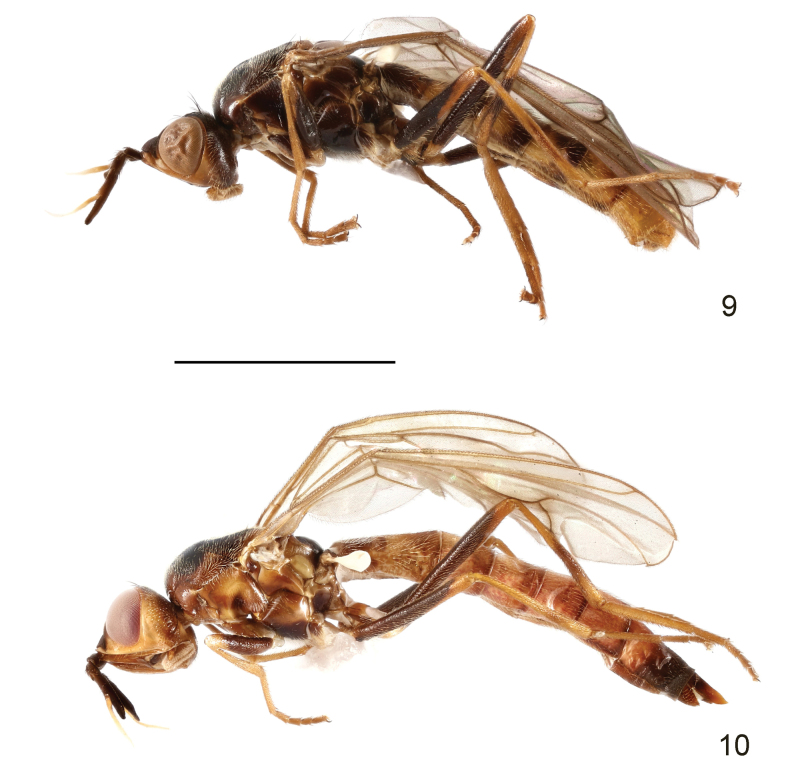
Loxocera (Loxocera) lonsdalei sp. nov., habitus, lateral **9** male, holotype **10** female, paratype. Scale bar: 3 mm.

**Figures 11–13.**
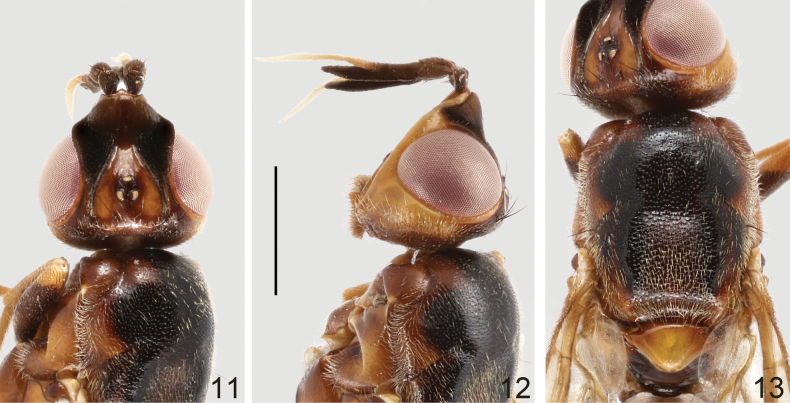
Loxocera (Loxocera) lonsdalei sp. nov., paratype, female **11** head, dorsal **12** same, lateral **13** thorax, dorsal. Scale bar: 1 mm.

**Male and female.** Body length 7.7–9.5 mm, wing length 6.0–6.5 mm, length of antenna 1.6–1.8 mm. Generally brown to blackish brown, moderately shining (Figs 9, 10). Frontal vitta black, with anterior part brown (Fig. 11); ocellar triangle and face yellowish brown; parafacial, gena and posterior eye margin dark yellow (Figs 11, 12). Antenna with arista whitish yellow (Fig. 12). Proboscis and palpus pale brown. Postpronotum brown to dark brown. Mesonotum largely blackish brown, with irregular brown to dark brown margin as shown in Fig. 13. Scutellum yellowish brown (Fig. 13). Mesopleuron dark yellow with variable brown to dark brown mottling (Fig. 10), or largely blackish brown (Fig. 9). Wing slightly infumated; wing veins yellowish brown to dark brown. Halter white with base slightly darkened. Legs dark brown to blackish brown; coxae brown to blackish brown; trochanters, apex of femora, tibiae, and tarsi yellowish brown; mid tibia with indistinct, narrow, dark ring subapically (Figs 9, 10); hind tibia with wide dark ring at middle (Figs 9, 10). Abdomen yellowish brown; anterior half and posterior margin of syntergite 1+2 and posterior portion of tergites 3–5 in male blackish brown (Fig. 9); anterior half and posterior margin of syntergite 1+2 and posterior portion of tergites 3–6 in female dark brown, posterior half of tergite 7 and anterior half of tergite 10 blackish brown (Fig. 10). Bristles on head and thorax black.

Head (Figs 11, 12) nearly rounded in dorsal view, largely glabrous; length along midline 0.9 times as long as width across eyes, width across eyes 2 times as broad as interocular space. Frons strongly protruding beyond level of anterior eye margin; frontal vitta with shallow depression at middle; frontal orbit with some short, scattered hairs. Ocellar triangle broad, smooth. Face strongly slanting, with weakly elevated median carina. Parafacial narrow, with a tomentose golden patch between anterior eye margin and lunule. Gena swollen; postgena covered with silvery tomentum. Occiput with a large silvery tomentose patch at middle above foramen. Head chaetotaxy: 1 ocellar seta, 1 postvertical seta, 1 inner vertical seta, 1 outer vertical seta. Antenna (Fig. 12) long and thick, with short dense setulae; pedicel distinctly longer than scape; first flagellomere about 3 times as long as pedicel, gradually narrowed towards apex; arista thin, arising near midpoint of first flagellomere, 1.3 times as long as first flagellomere, divided into small aristomere 1 and large aristomeres 2+3. Palpus elongate oval, with short dense golden setulae and long scattered black setae.

Thorax (Figs 9, 10, 13) robust, with short dense whitish yellow setulae, except anepisternum (anterior half), anepimeron, katatergite, katepisterum (middle portion), meron, scutellum and mediotergite (middle portion) glabrous; anatergite with fine tomentum. Scutum 1.2 times as long as wide. Scutellum (Fig. 13) subtriangular, slightly swollen and wider than long. Thoracic chaetotaxy: 1 dorsocentral seta, 1 notopleural seta, 2 posterior supra-alar setae, 1 apical scutellar seta. Wing with last sector of M_1_ strongly curved; apex of M_4_ nearly reaching wing margin. Legs with dense, whitish-yellow setulae, except ventral surface of fore and mid femora largely glabrous; femora subfusiform, slightly compressed laterally; tibiae gradually widened towards apex, nearly straight.

Abdomen elongate, with short, dense, whitish-yellow setae; syntergite 1+2 with several long hair-like setae laterally.

**Figures 14–17.**
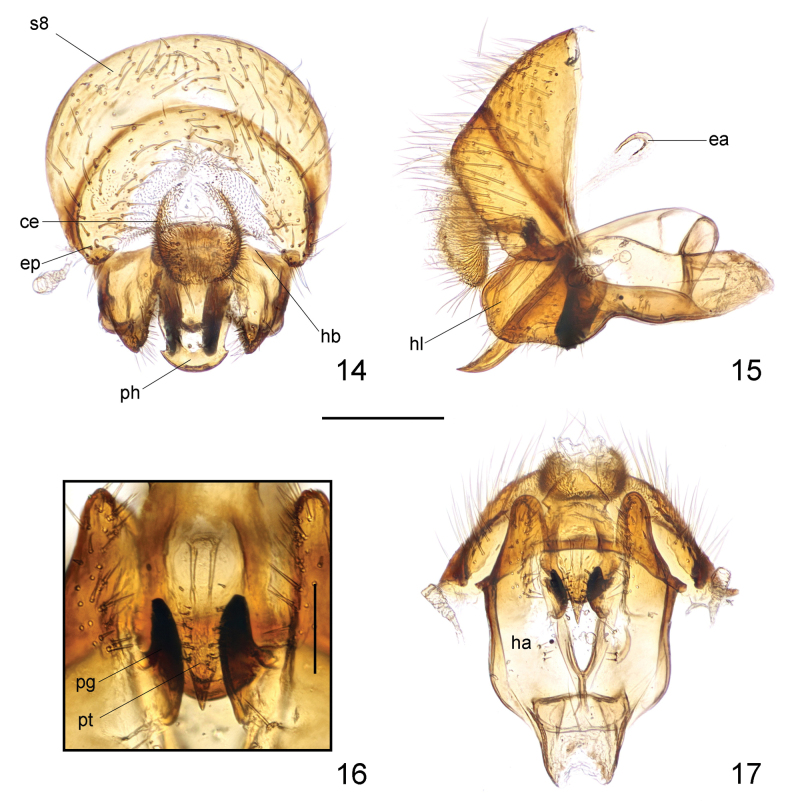
Loxocera (Loxocera) lonsdalei sp. nov., male genitalia **14** sternite 8 to genitalia, caudal **15** same, lateral **16** pregonite and phallus, ventral **17** hypandrium and associated structures, ventral. Abbreviations: ce = cercus, ea = ejaculatory apodeme, ep = epandrium, ha = hypandrial arm, hb = hypandrial bridge, hl = hypandrial lobe, pg = pregonite, s8 = sternite 8, ph = phallus, pt = phallotrema. Scale bars: 0.25 mm (**14, 15, 17**); 0.1 mm (**16**).

***Male genitalia***: Sternite 8 (Figs 14, 15) broad, relatively flattened, with long, dense setae. Cerci (Figs 14, 15) relatively slender, slightly curved, with short, dense setae. Hypandrium (Fig. 17) well developed; hypandrial arms posteriorly produced into very large, broad, convex lobes covered with short setae (Figs 14–17). Hypandrial bridge present and robust. Pregonite (Figs 14–16) short, simple in shape, apically blunt. Phallus (Figs 14, 15) shovel-like, relatively long, abruptly widened apically with rounded posterior margin. Phallotrema (Fig. 16) large, flanked by peculiar, short or long, simple or apically bifurcate processes. Ejaculatory apodeme (Fig. 15) small, hook-like, strongly curved.

***Female terminalia***: Segment 7 laterally compressed; sternite 7 keeled along midline. Segment 8 coriaceous and longitudinally striate, posterodorsal margin shallowly emarginate, posterolateral margin forming blunt angular protrusion, posteroventral margin with deep linear incision. Tergite 10 relatively long and broad, with tiny, scattered setulae. Cerci relatively broad, separate from tergite 10, with rows of long setulae on posterior margin.

##### Etymology.

The specific epithet is dedicated to the Canadian entomologist Owen Lonsdale (Agriculture and Agri-Food Canada, Ottawa, Canada), for his outstanding contribution to the systematics of Acalyptratae, and his encouragement to the first author.

##### Distribution.

China – Qinghai: Haibei, Haidong; Shaanxi: Xi’an (Fig. 37).

##### Comparative notes.

This new species is most similar to L. (L.) omei Shatalkin, 1998, but it can be easily distinguished from the latter by the following character states: antennal pedicel distinctly longer than scape [vs subequal in length in L. (L.) omei]; antennal first flagellomere distinctly narrowed towards apex [vs weakly narrowed in L. (L.) omei]; mesonotum largely blackish with irregular brownish margin [vs uniformly blackish in L. (L.) omei]; abdomen yellowish brown with distinct ring-like markings [vs blackish brown with apical segments reddish in L. (L.) omei].

Antennal morphology similar to that of this new species also presents in L. (L.) hoffmannseggi Meigen, 1826 from Central and West Europe and L. (L.) matsumurai Iwasa, 1992 from Japan. However, the coloration of head, thorax, and abdomen of the new species is very different from that of the latter two species. Additionally, the head of the new species is nearly rounded in dorsal view with the frons strongly protruding anteriorly, while in the latter two species, the head is transverse oblong in dorsal view and the frons weakly protrudes anteriorly.

#### ﻿ Loxocera (Loxocera) maculithorax sp. nov.

Taxon classificationAnimaliaDipteraPsilidae

34192CDF-A3FD-5445-A107-7E1D902DB407

https://zoobank.org/60A9A3DD-8E8A-4D3D-B507-9BB730CDC9F0

Figs 18–19 , 20–22 , 23–26

##### Type materials.

***Holotype*** (♂): China, Shaanxi, Baoji, Longxian, Guanshan Grassland, 2034 m, 2020.viii.13, leg. Bing Zhang (CAU). ***Paratypes***: same collection data as for holotype (1♂1♀, CAU).

##### Diagnosis.

Generally dark brown; face brown; antennal scape and pedicel subequal in length; apex of antennal first flagellomere produced beyond base of arista; arista laterally compressed and very high, arising at apex of antennal first flagellomere and 2.1 times as long as the latter; mesonotum blackish, with irregular, brownish margin; wing with broad, transverse, dark band at level of posterior crossvein; hypandrial lobe large, short, covered with long setae; pregonite stout, lobate, apically blunt, with some rounded processes; phallus tongue-like, relatively short, with rounded posterior margin.

##### Description.

**Figures 18, 19.**
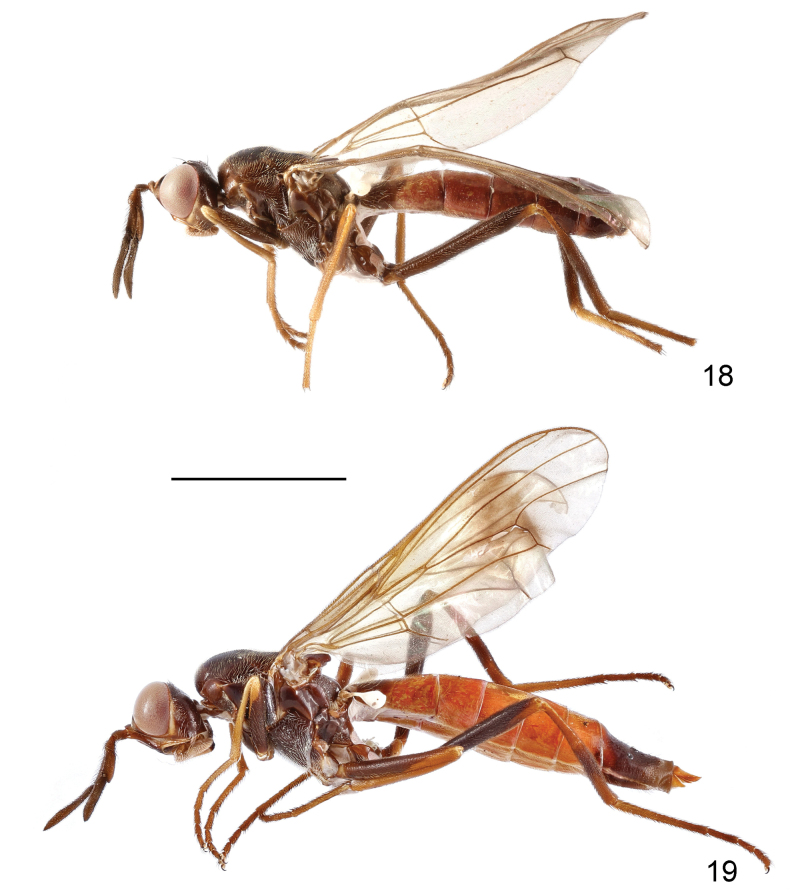
Loxocera (Loxocera) maculithorax sp. nov., habitus, lateral **18** male, holotype **19** female, paratype. Scale bar: 3 mm.

**Figures 20–22.**
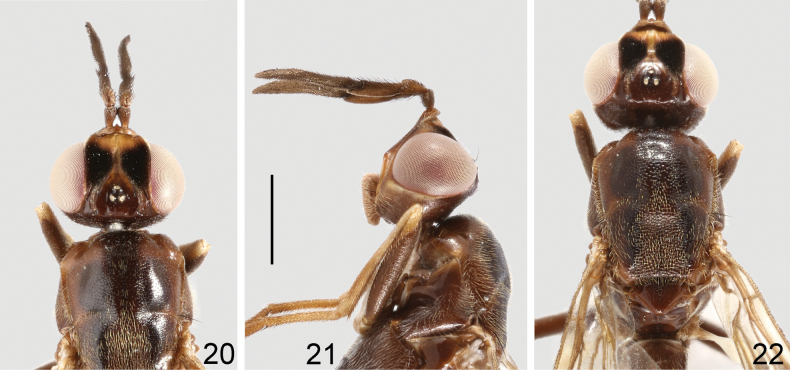
Loxocera (Loxocera) maculithorax sp. nov., holotype, male **20** head, dorsal **21** same, lateral **22** thorax, dorsal. Scale bar: 1 mm.

**Male and female.** Body length 8.8–10.5 mm, wing length 6.2–7.0 mm, length of antenna 1.9–2.3 mm. Generally dark brown, moderately shining (Figs 18, 19). Frontal vitta black, with anterior and median parts dark yellow (Fig. 20); face brown; parafacial, gena, postgena, proboscis, and palpus pale brown (Fig. 21). Postpronotum brown. Mesonotum largely blackish brown, with irregular, dark brown margin as shown in Fig. 22. Wing slightly infumated, with broad, transverse, dark band at level of posterior crossvein, band more or less interrupted along center of cells r_2+3_, r_4+5_ and dm; wing veins yellowish brown to brown. Halter white with base slightly darkened. Legs dark brown to blackish brown; trochanters, apex of femora, fore and mid tibiae, and tarsomere 1 yellowish brown; hind tibia in male dark brown except base and apex slightly paler (Fig. 18), in female yellowish brown, with wide, median, dark brown ring (Fig. 19). Abdomen reddish brown (♀, Fig. 19) to dark reddish brown (♂, Fig. 18); syntergite 1+2, tergite 6 and posterior portion of tergite 5 in male, and tergite 7 and posterior margin of tergite 6 in female blackish brown. Bristles on head and thorax black.

Head (Figs 20, 21) transverse oblong in dorsal view, largely glabrous; length along midline 0.7 times as long as width across eyes, width across eyes 2 times as broad as interocular space. Frons slightly protruding beyond level of anterior eye margin; frontal vitta with shallow depression at middle; frontal orbit with some short, scattered hairs. Ocellar triangle broad, smooth, with silvery tomentose stripes along lateral margins. Face strongly slanting, with moderately elevated median carina. Parafacial narrow, with a tomentose golden patch between anterior eye margin and lunule. Gena swollen; postgena covered with silvery tomentum. Occiput with a large silvery tomentose patch at middle above foramen. Head chaetotaxy: 1 ocellar seta, 1 postvertical seta, 1 inner vertical seta, 1 outer vertical seta. Antenna (Fig. 21) long and thick, with short, dense setulae; scape nearly as long as pedicel, gradually widened towards apex; first flagellomere about 2 times as long as pedicel, apex strongly curving ventrally, produced beyond base of arista; arista laterally compressed and very high, arising at apex of first flagellomere, 2.1 times as long as first flagellomere, divided into small aristomere 1 and large aristomeres 2+3. Palpus elongate oval, with short, dense setulae and long, scattered setae.

Thorax (Figs 18, 19, 22) robust, with short, dense, whitish-yellow setulae, except anepisternum (anterior half), anepimeron, katatergite, meron, scutellum, and mediotergite (middle portion) glabrous; anatergite with fine tomentum. Scutum 1.4 times as long as wide. Scutellum (Fig. 22) subtriangular, slightly swollen, and wider than long. Thoracic chaetotaxy: 1 dorsocentral seta, 1 notopleural seta, 2 posterior supra-alar setae, 1 apical scutellar seta. Wing with last sector of M_1_ strongly curved; apex of M_4_ nearly reaching wing margin. Legs with dense, whitish-yellow setulae, except ventral surface of fore and mid femora largely glabrous; femora subfusiform, slightly compressed laterally; tibiae gradually widened towards apex, fore and mid tibiae straight, hind tibia finely curved.

Abdomen elongate, with short, dense, whitish-yellow setae; syntergite 1+2 with several long hair-like setae laterally.

**Figures 23–26.**
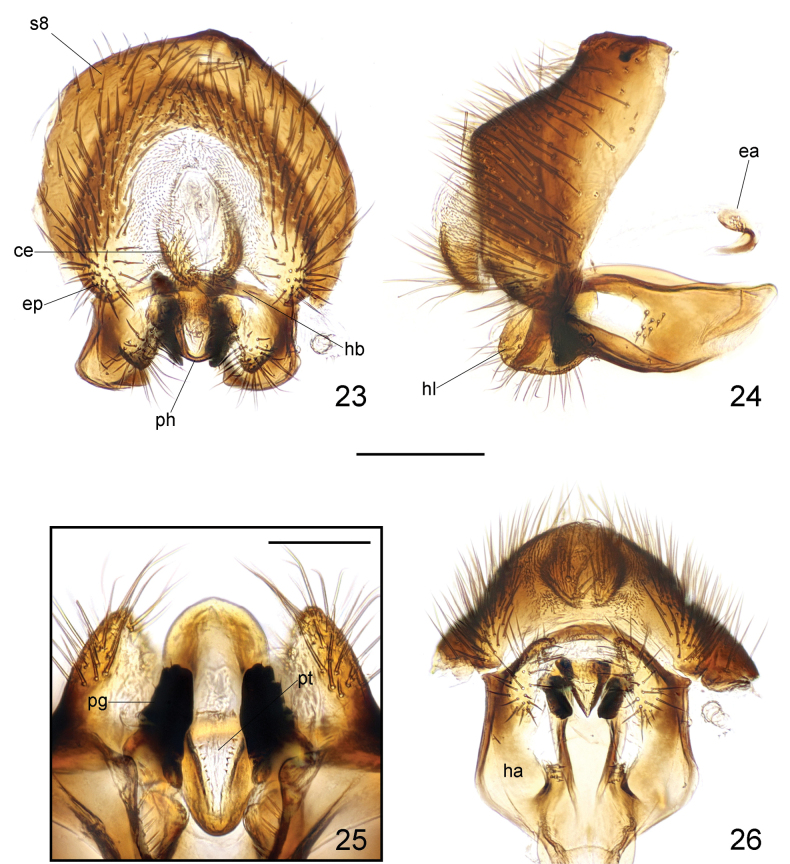
Loxocera (Loxocera) maculithorax sp. nov., male genitalia **23** sternite 8 to genitalia, caudal **24** same, lateral **25** pregonite and phallus, ventral **26** hypandrium and associated structures, ventral. Abbreviations: ce = cercus, ea = ejaculatory apodeme, ep = epandrium, ha = hypandrial arm, hb = hypandrial bridge, hl = hypandrial lobe, pg = pregonite, s8 = sternite 8, ph = phallus, pt = phallotrema. Scale bars: 0.25 mm (**23, 24, 26**); 0.1 mm (**25**).

***Male genitalia***: Sternite 8 (Figs 23, 24) broad, with long, dense setae, dorsally with a wide, blunt protrusion at middle. Cerci (Figs 23, 24) relatively broad, elongate, with short, dense setae. Hypandrium (Fig. 26) well developed; hypandrial arms fused posterodorsally, posteriorly produced into large, short, convex lobes covered with long setae (Figs 23–26). Hypandrial bridge present and robust. Pregonite (Figs 23–25) stout, lobate, apically blunt, with some rounded processes. Phallus (Figs 23–25) tongue-like, relatively short, with rounded posterior margin. Phallotrema (Fig. 25) large, flanked by some peculiar, tiny processes. Ejaculatory apodeme (Fig. 24) small, hook-like, strongly curved.

***Female terminalia***: Segment 7 laterally compressed; sternite 7 keeled along midline. Segment 8 coriaceous and longitudinally striate, posterodorsal margin shallowly emarginate, posterolateral margin forming acute angular protrusion, posteroventral margin with deep linear incision. Tergite 10 relatively short and narrow, with scattered tiny setulae. Cerci relatively broad, separate from tergite 10, with rows of long setulae on posterior margin.

##### Etymology.

The specific epithet is derived from Latin *macula* (meaning spotted) and Greek *thorax* (meaning thorax), referring to the mesonotum of the new species, which is blackish with an irregular brownish margin.

##### Distribution.

China – Shaanxi: Baoji (Fig. 37).

##### Comparative notes.

This new species is similar to L. (L.) fujiana, but it can be readily separated from the latter by the different coloration of head, mesonotum, hind tibia, and abdomen, the apically curved and produced first flagellomere, and the shortened scutum. It also resembles L. (L.) chikuni sp. nov., and their differences are discussed above [see under L. (L.) chikuni sp. nov.]. The Japanese species L. (L.) monstrata Iwasa, 1992 shares a similar antennal morphology with the new species, but its arista is less than 2 times as long as the first flagellomere, and it has different coloration on thorax, legs and abdomen.

#### ﻿ Loxocera (Loxocera) obscura sp. nov.

Taxon classificationAnimaliaDipteraPsilidae

49EF586B-FEA2-5461-97B1-6EE1B91E16F8

https://zoobank.org/30ECC9F7-08E8-4782-90F9-F305B414E095

Figs 27–30 , 31–34

##### Type materials.

***Holotype*** (♂): China, Shaanxi, Xi’an, Zhouzhi, Houzhenzi, 2009.ix.29, leg. Maoling Sheng (CAU). ***Paratypes***: same collection data as for holotype (4♂♂, CAU).

##### Diagnosis.

Generally dark brown; face blackish; antennal scape distinctly longer than pedicel; antennal first flagellomere stick-like, weakly narrowed towards apex of segment; arista whitish yellow, thin, arising before midpoint of antennal first flagellomere and 1.4 times as long as the latter; wing without transverse dark band; hypandrial lobe very large, broad, covered with short, sparse setae on inner surface; pregonite rather long, slender, curved apically with sharp apex; phallus droplet-like, elongate, abruptly narrowed apically.

##### Description.

**Figures 27–30.**
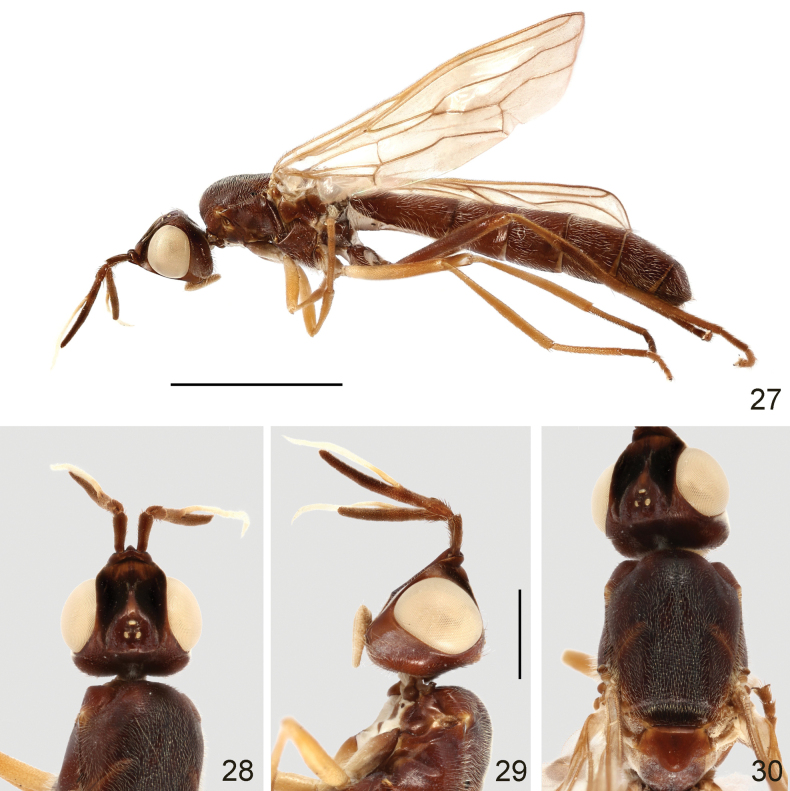
Loxocera (Loxocera) obscura sp. nov., holotype, male **27** habitus, lateral **28** head, dorsal **29** same, lateral **30** thorax, dorsal. Scale bars: 3 mm (**27**); 1 mm (**28–30**).

**Male.** Body length 8.9–10.2 mm, wing length 6.6–7.2 mm, length of antenna 2.2–2.5 mm. Generally dark brown, moderately shining (Fig. 27). Frontal vitta black except anterior and median parts brown (Fig. 28); face blackish brown; gena and postgena slightly paler (Fig. 29). Antenna with arista whitish yellow. Proboscis and palpus pale brown. Mesonotum blackish brown (Fig. 30). Scutellum brown (Fig. 30). Mesopleuron with anepimeron and katatergite slightly paler; portion above and below anterior spiracle yellow (Fig. 29). Wing slightly infumated; wing veins yellowish brown to dark brown. Halter white with base slightly darkened. Fore and mid legs dark yellow, with coxae dark brown, base of femora pale yellow, and mid tarsomeres 2–5 slightly darkened. Hind leg brown, with coxa, femur (except apex), and wide median ring on tibia dark brown. Bristles on head and thorax black.

Head (Figs 28, 29) nearly rounded in dorsal view, largely glabrous; length along midline nearly as long as width across eyes, width across eyes 2 times as broad as interocular space. Frons strongly protruding beyond level of anterior eye margin; frontal vitta with shallow depression at middle; frontal orbit with some short, scattered hairs. Ocellar triangle broad, smooth. Face strongly slanting, with moderately elevated median carina. Parafacial narrow, with a tomentose golden patch between anterior eye margin and lunule. Gena swollen; postgena covered with silvery tomentum. Occiput with a large silvery tomentose patch at middle above foramen. Head chaetotaxy: 1 ocellar seta, 1 postvertical seta, 1 inner vertical seta 1 outer vertical seta. Antenna (Fig. 29) long, relatively slender, with short, dense setulae; scape distinctly longer than pedicel; first flagellomere about 4.6 times as long as pedicel, stick-like, laterally compressed, weakly narrowed towards apex; arista thin, arising before midpoint of first flagellomere, 1.4 times as long as first flagellomere, divided into small aristomere 1 and large aristomeres 2+3. Palpus elongate oval, with short, dense, white setulae.

Thorax (Figs 27, 30) robust, with short, dense, white setulae, except anepisternum (anterior half), anepimeron, katatergite, meron, scutellum and mediotergite (middle portion) glabrous; anatergite with fine tomentum. Scutum 1.35 times as long as wide. Scutellum (Fig. 30) transverse, wider than long, with midportion distinctly swollen. Thoracic chaetotaxy: 1 dorsocentral seta, 1 notopleural seta, 2 posterior supra-alar setae, 1 apical scutellar seta. Wing with last sector of M_1_ strongly curved; apex of M_4_ nearly reaching wing margin. Legs with dense, whitish-yellow setulae, except ventral surface of fore and mid femora largely glabrous; femora subfusiform, slightly compressed laterally; tibiae gradually widened towards apex, hind tibia finely curved.

Abdomen elongate, with short, dense, white setae; syntergite 1+2 with several long, hair-like setae laterally.

**Figures 31–34.**
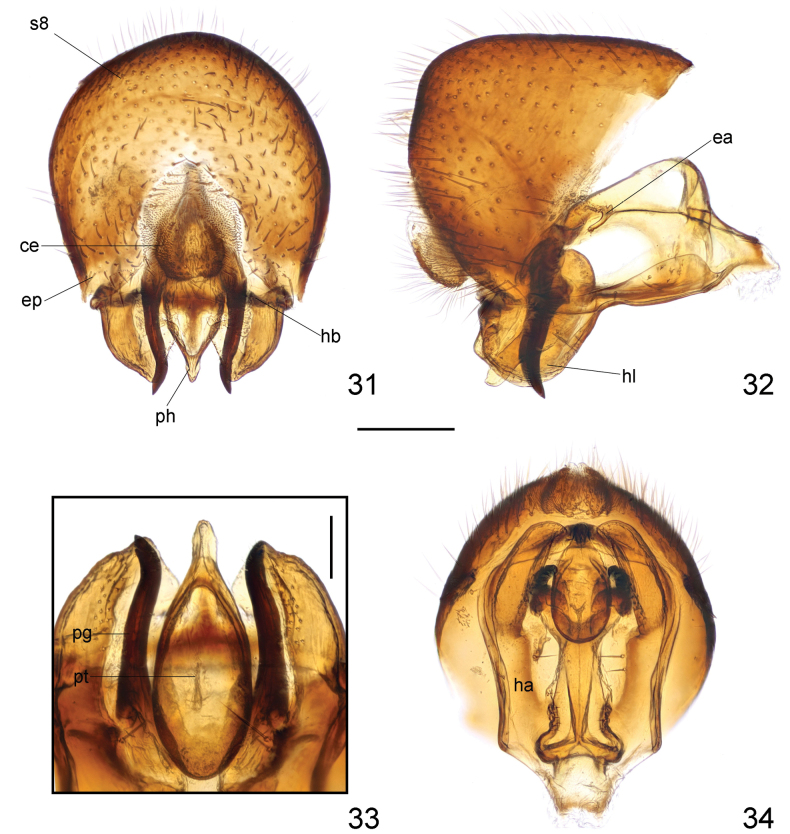
Loxocera (Loxocera) obscura sp. nov., male genitalia **31** sternite 8 to genitalia, caudal **32** same, lateral **33** pregonite and phallus, ventral **34** hypandrium and associated structures, ventral. Abbreviations: ce = cercus, ea = ejaculatory apodeme, ep = epandrium, ha = hypandrial arm, hb = hypandrial bridge, hl = hypandrial lobe, pg = pregonite, s8 = sternite 8, ph = phallus, pt = phallotrema.Scale bars: 0.25 mm (**31, 32, 34**); 0.1 mm (**33**).

***Male genitalia***: Sternite 8 (Figs 31, 32) broad, inflated, with long, dense setae. Cerci (Figs 31, 32) relatively broad, slightly curved, with short, dense setae. Hypandrium (Fig. 34) well developed; hypandrial arms posteriorly produced into very large, broad, convex lobes covered with sparse, short setae on inner surface (Figs 31–34). Hypandrial bridge present and robust. Pregonite (Figs 31–33) rather long, slender, curved apically with sharp apex. Phallus (Figs 31–33) droplet-like, elongate, abruptly narrowed apically. Phallotrema (Fig. 33) small, without processes. Ejaculatory apodeme (Fig. 32) small, V-like, strongly curved.

**Female.** Unknown.

##### Etymology.

The specific epithet is derived from Latin *obscura* (meaning dark, indistinct), referring to the dark-brown body color of the new species.

##### Distribution.

China – Shaanxi: Xi’an (Fig. 37).

##### Comparative notes.

This new species is most similar to L. (L.) malaisei (Frey, 1955) (from Myanmar and Nepal) in having a relatively long antennal scape (longer than pedicel), a nearly parallel-sided and laterally compressed antennal first flagellomere, similar coloration of legs, and an enlarged and posteriorly inflated male sternite 8. It can be distinguished from the latter by the following character states: antennal scape about 1.5 times as long as pedicel [vs 2 times in L. (L.) malaisei]; antennal first flagellomere elongate, 4.6 times as long as pedicel [vs shorter, 4 times in L. (L.) malaisei]; arista slender [vs widened towards apex in L. (L.) malaisei]; phallus droplet-like with apex abruptly narrowed [vs elongate oval in L. (L.) malaisei].

#### ﻿ Loxocera (Loxocera) omei

Taxon classificationAnimaliaDipteraPsilidae

Shatalkin, 1998

10706BA5-0EDD-5D77-A26A-5E7F08AE8A6F

Figs 35 , 36

Loxocera (Platystyla) omei Shatalkin, 1998: 90, 97 (protologue). Holotype (♂): China, Sichuan, Emeishan, USNM.
Loxocera (Loxocera) omei : Buck and Marshall (2006b: 199) (subgeneric placement, distribution).
Loxocera omei : Tang et al. (2021: 234) (catalogue, distribution).

##### Type material examined.

***Holotype*** (♂): China, Sichuan, Leshan, Emeishan, 1935.vii.21, leg. D.C. Graham (USNM).

##### Diagnosis.

Generally blackish brown; face yellowish brown; antennal scape and pedicel subequal in length; antennal first flagellomere laterally compressed, about 3.6 times as long as pedicel, weakly narrowed towards apex; arista whitish yellow, thin, arising near midpoint of antennal first flagellomere and 1.3 times as long as latter; wing without transverse dark band.

##### Distribution.

China – Sichuan: Leshan (Fig. 37).

##### Remarks.

**Figures 35, 36.**
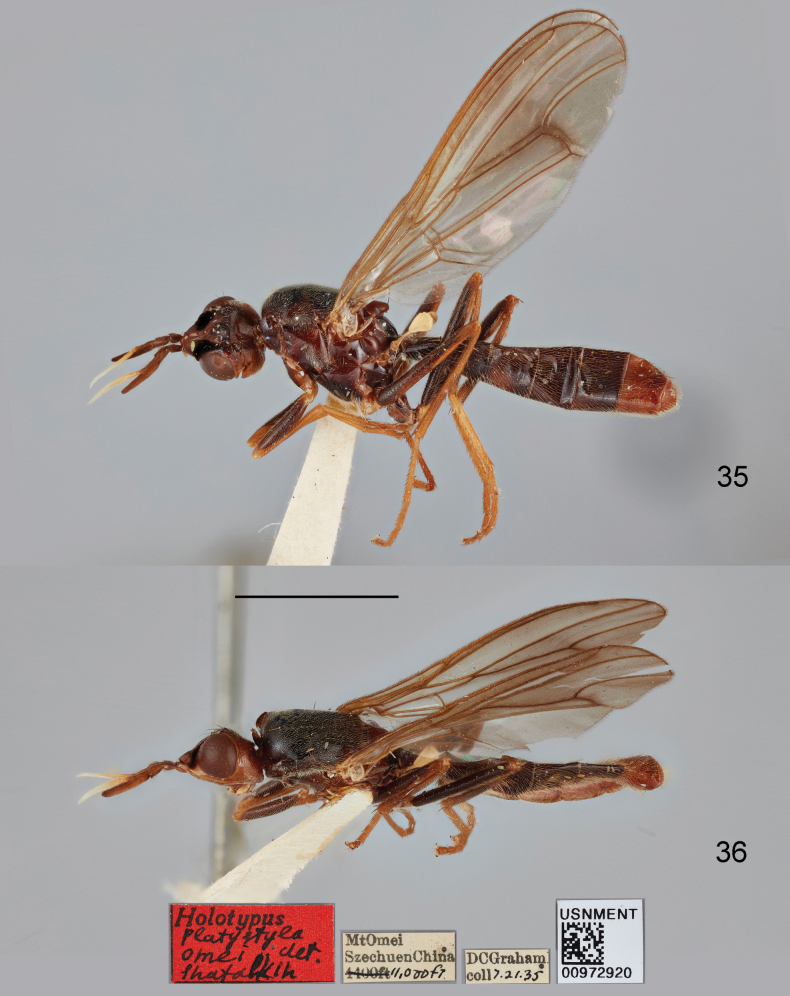
Loxocera (Loxocera) omei Shatalkin, 1998, holotype, male, habitus with labels **35** lateral **36** dorsal. Scale bar: 3 mm.

This species was described based on one male (the holotype) from Sichuan, China (Shatalkin 1998). The habitus photographs of the holotype (Figs 35, 36) are provided here for facilitating the identification of this species.

**Figure 37.**
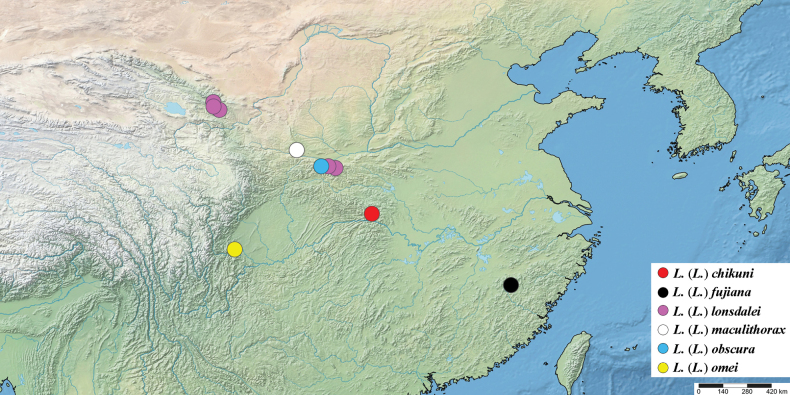
Known distribution of species of the subgenus Loxocera in China.

### ﻿Notes on species transferred to *Imantimyia* Frey, 1925

#### ﻿ Loxocera (Imantimyia) anulata

Taxon classificationAnimaliaDipteraPsilidae

Wang & Yang, 1998

CF9D31DE-3A55-5C37-BAB5-22C3F340C17A

Figs 38 , 39

Loxocera (Loxocera) anulata Wang and Yang 1998a: 440, 454 (protologue). Holotype (♂): China, Hubei, Shennongjia, CAU.
Loxocera annulata : Buck and Marshall (2006b: 199) (listed, distribution). Incorrect subsequent spelling.
Loxocera anulata : Tang et al. (2021: 234) (catalogue, distribution).

##### Type material examined.

***Holotype*** (♂): China, Hubei, Shennongjia, Dajiuhu, 1977.vii.9, leg. Huanguang Zou (CAU).

##### Distribution.

China – Hubei: Shennongjia.

##### Remarks.

*Loxoceraanulata* was originally described as a member of the subgenus Loxocera based on one male (the holotype) from Hubei, China (Wang and Yang 1998a). The specific epithet of this species was spelled as *anulata* in its original description, where the authors (Wang and Yang 1998a) intend to create the name from the Latin adjective *anulatus*, -*a*, -*um* (meaning ringed, referring to the annulations on the abdomen of this species), therefore it is considered as the correct original spelling. The subsequent usage of the specific epithet *annulata* (Buck and Marshall 2006b) is treated as an incorrect subsequent spelling. Buck and Marshall (2006b) failed to assign this species (as *L.annulata*) to a certain subgenus based on its brief original description. This species was catalogued by Tang et al. (2021) without subgeneric placement.

**Figures 38, 39.**
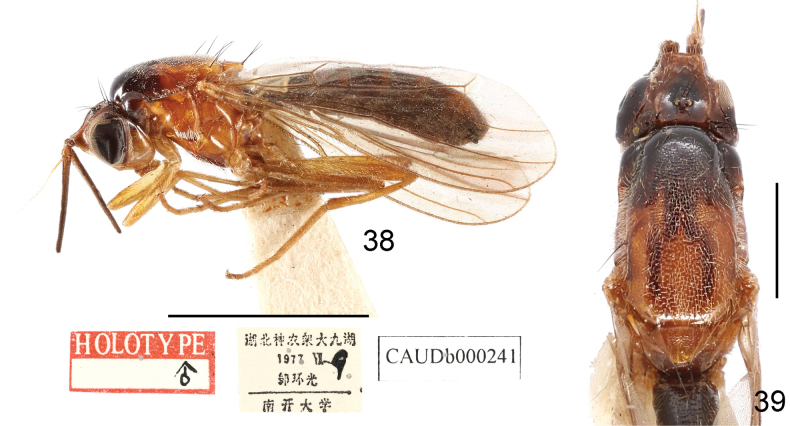
Loxocera (Imantimyia) anulata Wang & Yang, 1998, holotype, male **38** lateral habitus with labels **39** head and thorax, dorsal. Scale bars: 3 mm (**38**); 1 mm (**39**).

The holotype of *L.anulata* was examined during the present study. It satisfied the diagnosis of *Loxocera* s. lat. provided by Buck and Marshall (2006b). The sclerotized and sub-shining frontal vitta, the hiding lunule, the microtrichose on the alula, and the very reduced and bare male sternite 8 match the characters used to diagnose the subgenus Imantimyia Frey, 1925 (Buck and Marshall 2006b). Therefore, this species is herein transferred to *Imantimyia*.

#### ﻿ Loxocera (Imantimyia) tianmuensis

Taxon classificationAnimaliaDipteraPsilidae

Wang & Yang, 1998

FB593904-DB82-5E76-BA1E-54ED6FA96D40

Figs 40 , 41

Loxocera tianmuensis Wang and Yang 1998b: 200, 201 (protologue); Buck and Marshall (2006b: 199) (listed, distribution). Holotype (♂): China, Zhejiang, Xitianmushan, CAU.

##### Type material examined.

***Holotype*** (♂): China, Zhejiang, Lin’an, Xitianmushan, 350 m, 1987.ix.3, leg. Qun Ma (CAU).

##### Distribution.

China – Zhejiang: Lin’an.

##### Remarks.

*Loxoceratianmuensis* was originally described based on one male (the holotype) from Zhejiang, China (Wang and Yang 1998b), but the authors did not assign it to any subgenus. Buck and Marshall (2006b) mentioned that this species “cannot be confidently placed to subgenus” due to the inadequate original description. No new information has since been published on this species.

**Figures 40, 41.**
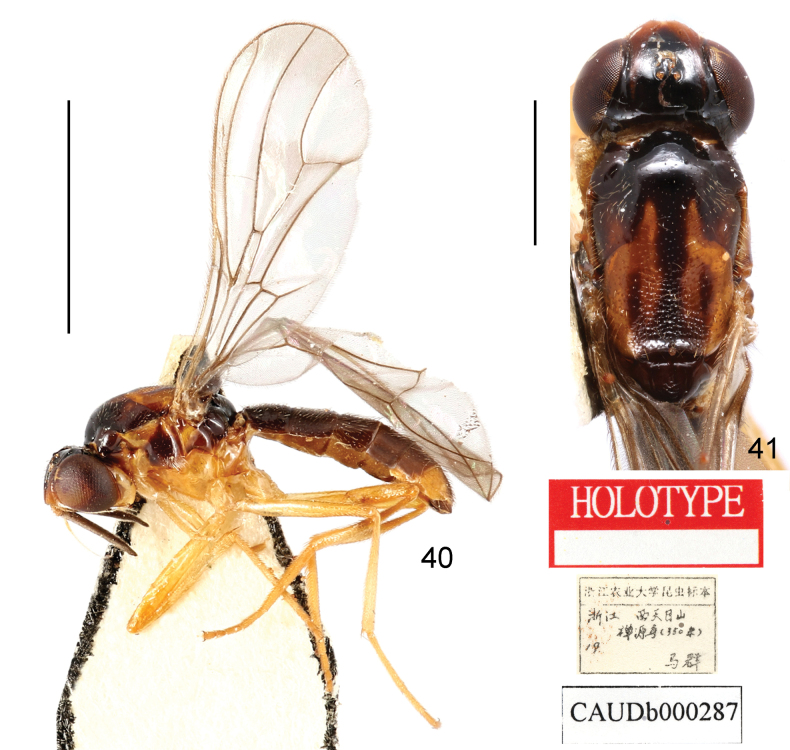
Loxocera (Imantimyia) tianmuensis Wang & Yang, 1998, holotype, male **40** lateral habitus with labels **41** head and thorax, dorsal. Scale bars: 3 mm (**40**); 1 mm (**41**).

The holotype of *L.tianmuensis* was examined in the course of the present study. The date on the collection data label of the holotype does not match that in the original description. The authors (Wang and Yang 1998b) communicated personally with the specimen collector during the preparation of the manuscript, thus providing a precise date of specimen collection in the original description. Based on the examination of the holotype, this species matches the current concept of *Imantimyia* (Buck and Marshall 2006b) and is herein placed under this subgenus.

## ﻿Discussion

The present study documents the Chinese fauna of the subgenus Loxocera, including two described species and four new species. The other two species originally assigned to the subgenus Loxocera are herein transferred to the subgenus Imantimyia based on examination of their holotypes. An identification key to the species of the subgenus Loxocera from China is also presented.

Antennal morphology of members of the subgenus Loxocera is diverse and useful in species identification (Zhou et al. 2022). The arista is particularly highly diagnostic, with half of the fauna having the atista dark colored, laterally compressed, very high and arising at the apex of the antennal first flagellomere, and the other half having it whitish yellow, slender, and arising near the midpoint of the antennal first flagellomere. The location and shape of the arista have been used by some authors to distinguish genus-level taxa within *Loxocera* s. lat. (e.g. Hennig 1941; Steyskal 1987; Iwasa 1992; Shatalkin 1998; Shatalkin and Merz 2010), while others consider it more appropriate for species-level identification (Buck and Marshall 2006b; Zhou et al. 2022). Whether these states characterize natural species groups remains to be proven.

Including the four species newly described in this study, the subgenus Loxocera currently comprises 14 species (Table 1). Many of these species are distributed in the Palaearctic Realm, while only three are reported from the northern part of the Oriental Realm. Among the six species currently recorded in China, only L. (L.) fujiana occurs in the Oriental Realm. Most of the Chinese species of the subgenus Loxocera are endemic and known only from their type locality, except for L. (L.) lonsdalei sp. nov. which has been collected from four localities in central China. Considering the diversity presented from so few specimens, it is very likely that many more undescribed species will be found given additional collecting.

**Table 1.** Described species of the subgenus Loxocera and their known distribution.

Species	Distribution	References
L. (L.) aristata (Panzer, 1801)	Europe, Iran, Israel	Panzer 1801; Freidberg and Shatalkin 2008; Khaghaninia and Gharajedaghi 2014; Whiters and Claude 2021
L. (L.) atriceps Bigot, 1886	Europe	Bigot 1886; Buck and Marshall 2006b
L. (L.) chikuni Zhou & Yang, sp. nov.	China	present study
L. (L.) fujiana (Wang, 1999)	China	Zhou et al. 2022; present study
L. (L.) glandicula Iwasa, 1993	Nepal	Iwasa 1993
L. (L.) hoffmannseggi Meigen, 1826	Europe	Whiters and Claude 2021
L. (L.) lonsdalei Zhou & Yang, sp. nov.	China	present study
L. (L.) maculata Rondani, 1876	Europe	Rondani 1876; Soós 1984; Buck and Marshall 2006b
L. (L.) maculithorax Zhou & Yang, sp. nov.	China	present study
L. (L.) malaisei Frey, 1955	Myanmar, Nepal	Frey 1955; Iwasa 1993
L. (L.) matsumurai Iwasa, 1992	Japan, Russia	Iwasa 1992
L. (L.) monstrata Iwasa, 1992	Japan	Iwasa 1992
L. (L.) obscura Zhou & Yang, sp. nov.	China	present study
L. (L.) omei Shatalkin, 1998	China	Shatalkin 1998; present study

## Supplementary Material

XML Treatment for Loxocera

XML Treatment for Loxocera (Loxocera) chikuni

XML Treatment for Loxocera (Loxocera) fujiana

XML Treatment for Loxocera (Loxocera) lonsdalei

XML Treatment for Loxocera (Loxocera) maculithorax

XML Treatment for Loxocera (Loxocera) obscura

XML Treatment for Loxocera (Loxocera) omei

XML Treatment for Loxocera (Imantimyia) anulata

XML Treatment for Loxocera (Imantimyia) tianmuensis

## ﻿Additional information

### Author ORCIDs

Jiale Zhou https://orcid.org/0000-0002-8901-3187

Ding Yang https://orcid.org/0000-0002-7685-3478

### Data availability

All of the data that support the findings of this study are available in the main text.
